# Role of maternal and child health services on the uptake of contraceptive use in India: A reproductive calendar approach

**DOI:** 10.1371/journal.pone.0269170

**Published:** 2022-06-15

**Authors:** Anjali Bansal, P. Shirisha, Bidhubhusan Mahapatra, Laxmi Kant Dwivedi

**Affiliations:** 1 International Institute for Population Sciences, Govandi East, Mumbai, India; 2 Department of Humanities and Social Sciences, Humanities and Science Block, IIT Madras, Chennai, Tamil Nadu, India; 3 Population Council, New Delhi, India; University of Salamanca, SPAIN

## Abstract

**Background:**

According to the latest round of National Family Health Survey—4 (NFHS (2015–16)) maternal and child health care (MCH) services improved drastically compared to NFHS-3. Previous studies have established that the uptake of MCH services increases the likelihood of early adoption of contraceptives among women. So, our study aims to examine if the early initiation of contraceptive has proportionately improved with the recent increase in MCH services.

**Methods:**

This study used the reproductive calendar of NFHS-4, 2015–16, to evaluate contraceptive initiation within 12 months after the last birth among 1,36,962 currently married women in India. A complementary log-log regression model was created to examine the link between the time of initiation of contraception and MCH care at the national level.

**Results:**

It was found that only a quarter of women within 12 months from last birth have adopted the modern contraceptive method. Among those majority of the females adopted sterilization mostly at the time of birth. The multivariable model identified, that the period of initiation of contraceptive depends on the gender composition of children and access to MCH services. It was found that the odds of early initiation of contraceptive use was higher when a women have only son (AOR = 1.15,95% CI– 1.22, 1.18) compared to women with only daughter. Also, it was found that women who have availed MCH services were more likely to adopt contraceptives earlier.

**Conclusion:**

The number of women availing MCH services has increased in India, but it did not result in a proportional increase in initiation of contraception after childbirth. Facilitating family planning services alongside MCH services will be beneficial in low-resource settings. It is a golden opportunity to educate and encourage women for early adoption of contraceptive.

## Introduction

Postpartum Family Planning (PPFP) is defined as the prevention of closely spaced and unwanted pregnancies in the first 12 months following childbirth [1]. Though women become fecund before the resumption of menses, many don’t initiate contraception [2]. This increases the risk of unintended pregnancy [3, 4]. Postpartum contraceptive initiation plays important role in reducing both maternal and infant mortality [1, 5]. PPFP varies by different demographic characteristics such as age, socioeconomic status, education level, residence, marital status [6, 7]. Also, the risk of maternal deaths could be two times higher among those who don’t use contraceptives than users [8]. Proper birth spacing reduces maternal and infant mortality risk factors like anaemia, and third trimester bleeding [9]. Also, if the birth span between the two subsequent births is longer, women can allocate more time for postnatal care like breastfeeding and proper nutrition [10]. Despite the likelihood of returning to fertility before the resumption of menses, women estimate pregnancy chances to be minimal when they are amenorrhoeic following birth and during breastfeeding. Breastfeeding was the most common reason for the non-use of contraceptive among postpartum women [11–14]. Experience with the contraceptives has also been demonstrated to be an important predictor of postpartum contraceptive use in studies. Women who had used contraceptives in the past were more likely to use contraceptives in the postpartum period. While women who had a poor experience in the past were reluctant to use it again. The barriers to PPFP were mostly associated with the fear associated with the utilisations of contraceptives- affecting the milk supply, excessive bleeding, migraines, injury, pain, discomfort as well as an inconvenience associated with IUD insertion [6, 12, 13, 15, 16]. A study from India found spousal violence and lack of marital communication to be barriers for the usage of contraceptives in the postpartum period [17]. Issues of the inconsistency in supply, stock out, and hindrance in accessibility as well as lack of awareness related to the contraceptives methods were also reported by some studies as barriers. Some religious norms too prohibit the use of contraceptive and encourage childbearing explained the regional differences in the use of contraceptive. Certain facility-based factors were found to be strongly associated with the PPFP such as institutional delivery, ANC, PNC [18]. Counselling for contraceptives during MCH care has been found to be an important predictor and was found to be more effective if conducted during both ANC and PNC visits. A study from India found that counselling should ideally begin during the prenatal period and involve discussions started by the practitioner. Patients can be guided to choose an effective and appropriate method of contraceptive with a little time and effort with the physician by their side [19].

The study findings of influence of MCH services on subsequent contraceptive has important policy implications [20]. As, in India 84% of women have visited the MCH centre at least once to receive some services [9] which provides an opportunity to educate and motivate women to initiate contraception use just after the delivery, and providing all the information in the same place can prove to be cost-effective [21]. The government has also launched *Janani Suraksha Yojana*, *Janani Shishu Suraksha Karyakram*, Pradhan Mantri *Surakhshit Matritva Abhiyan* to provide quality MCH services [22]. These points of contact are potential opportunities for providers to screen, counsel, and address the contraceptive needs of postpartum women [23].

Studies have shown that MCH services can be ’gateways’ to promote contraceptive use [9, 20, 24], but only a handful of studies have analysed MCH service utilisation and its impact on the timing of contraceptive initiation. A study in India found that an increase in the utilization of MCH services increases the use of family planning services which in turn paves path for safe motherhood and child survival [25]. Another study along similar lines found that integrating postpartum services with MCH services like antenatal and postnatal visits provided an opportunity for initiating contraceptive use [26]. Dixit, Dwivedi, and Gupta explored the early initiation of contraception, and its association with MCH services, which was very low in 2005–06 [9]. Given the significant association and considerable improvement in the use of MCH services reported by NFHS-4 (2015–16). This study hypothesizes that exposure to MCH services would increase the likelihood of early initiation of contraceptive use among currently married women.

Our intent is to understand whether MCH services impact the subsequent contraceptive use within the 12 months from the birth of the last child, using the reproductive calendar. The study period is restricted to 12 post-pregnancy periods because a mother and child have a higher mortality risk during this period [27]. Women contact service providers while accessing these MCH services, instilling trust in the healthcare system. Promoting contraceptive use during each contact in the continuum of MCH care could be considered an essential strategy for addressing the widespread unmet needs in family planning and timely intervention could reduce the unmet needs for family planning and thereby averting several unwanted pregnancies. Hence, our study findings could have important policy implications for the policymakers and stakeholders who aim to improve the contraceptive uptake in India.

## Material and methods

### Ethics statement

NFHS-4 survey has been conducted by the International Institute for Population Sciences (IIPS) under the aegis of MoHFW. The approval for NFHS-4 was obtained from the Ethics Review Board of the IIPS, Mumbai, India and ICF International Review Board (IRB). In this survey, written consent was obtained from the participants before the commencement of the interview. Each participants’ approval was sought, and then only interviews were conducted. Confidentiality was maintained as NFHS-4 published gathered data with no identifiable information of the survey participants. NFHS-4 is the anonymous, publicly available dataset, and is accessible upon request from the DHS Program at https://dhsprogram.com/data/available-datasets.cfm.

### Data

The data used for the analysis is the fourth round of the NFHS-4 (2015–2016). It is a nationally representative cross-sectional survey which includes representative sample of the households throughout India. The survey provides state, national and district level estimates of demographic, health as well as socio-economic status and program dimensions, which are critical for implementing the desired changes in demographic and health parameters. Stratified, two-stage sampling is mostly used in all DHS surveys to obtain a representative sample of households. Probability proportional to size (PPS) was used to select the households from all states and Union Territories. Within each rural stratum, villages were selected from the sampling frame with PPS. In urban areas, Census Enumeration block (CEB) were selected based on data from the census of India (the detailed sampling method is available here [28]). The survey for the first time in 2015–2016 provided data on key indicators associated with the demographic and health parameter at district level.

### Analytical sample

The study used the contraceptive calendar to assess the time of modern contraceptive use after delivery of the last child. At the time of the survey, the contraceptive behaviour and fertility of each woman or her husband were assessed at regular time intervals (month-by-month basis) in the contraceptive calendar. NFHS also collects MCH related aspects, like complete antenatal care, delivery care-institution details, postnatal care, immunization of child, etc. The outcome variable was the timing of contraceptive initiation following childbirth versus non-use of contraceptives with censoring. Censoring is a condition where the value of the unit of analysis is only partially known. In our research, we haven’t included the left-censored cases before the start of the calendar.

### Sample

The NFHS fourth round collected data from 6,99,686 ever-married women aged 15–49 in 6,01,509 households. Since the objective was to examine initiation of contraceptive use after the last birth within 12 months, so the sample was restricted to 1,36,962 currently married women.

### Study variables

#### Outcome variable

The contraceptive calendar contains information of all the reproductive event for the last five years prior to the survey (60 months). Since the information was collected in a string format, the position of last birth was extracted from that string. After extracting the position of last birth, the calendar was restricted to initiation of contraceptive methods within 12 months after the last childbirth. The modern contraceptive methods are sterilization, injectable, intrauterine devices, pills, condoms, diaphragm, foam/jelly, and other modern methods.

#### Explanatory variables

Background variables of the study are the place of residence, geographic region, caste, religion, wealth index, age of women, educational status of women, relevant knowledge, number of dead children, gender composition of living children, desire for another child, and husband’s desire for more children. The list of categorized independent variables is in S1 Table. All the variables are recoded as described in the S1 Table.

To identify the relationship between utilization of MCH care and contraceptive initiation time, we constructed an MCH index using the following MCH care variables

1. The number of Antenatal Care (ANC) visits (At least 4 visits).
2. Postnatal Care (PNC) of the mother (within 41 days after the delivery)
3. Number of Tetanus injections received during pregnancy (maximum 2 doses).
4. DPT-3 immunization received.
5. Institutional delivery by trained professionals.
6. Consumption of 100 + Iron/Folic Acid (IFA) Tablets.

First, all the variables were recoded as dichotomous, 0 and 1. ANC visits was recoded as 0 if women had ANC visit less than 4, and 1 if ANC visit more than equal to 4. PNC of mother was recoded as 0 if women had no PNC care or care after 41 days of delivery, and 1 if PNC within 41 days of delivery. Number of TT injections received during the pregnancy was recoded as 0 if women received less than 2 TT injections, and 1 otherwise. DPT3 immunization was recoded as 0 if women had received no DPT3 immunization for her last child or 1 otherwise. Institutional delivery was recoded as 0 if women delivered at home, and 1 if women delivered in health facility (both public and private). If women have taken 100+ IFA tablets, the variable was recoded as 1, and 0 otherwise. Principal component analysis (PCA) was then run based on the latter variable to create a MCH index, which is in continuous scale. The high value of MCH index indicates high uptake of MCH services and vice versa.

### Statistical analysis

We used a discrete-time analysis approach as the period for analysis in the recent survey was recorded in the nearest completed months. When the survival time data was analysed, few ties were observed because, at times, two or more women started using contraceptives simultaneously, which results in bias if the partial likelihood estimation is used [29]. The use of hazard rate as a variable is inappropriate in this case as it cannot be bound by 0 and 1. So it is essential to convert it into an appropriate scale that is freely bound as an independent variable [30]. A complementary log-log model is the most appropriate instrument in this situation. The unique advantage of the complementary model is that it can be used when data is asymmetrical in [0, 1] interval, where the logit and probit model are not appropriate. Therefore, a complementary log-log link function has been used after adopting the discrete-time approach. Data analysis was performed using Stata software version 15.0. Multivariable complementary log-log model adjusted odds ratio and 95% confidence interval associated with modern contraceptive method use among women were reported. A p-value of less than 5% was considered significant. Since confidence intervals are affected by the complex sampling design, we took into account the complex survey design of DHS within the svyset and svy procedures in Stata [31]. For the descriptive statistics, we have used women’s individual weight (v005) to produce the weighted frequencies, and percentages.

## Results

The descriptive statistics of the explanatory variables were presented in S2 Table. Table 1 presents the percentage distribution of initiation of different contraceptive methods by women in India within the first 12 months of postpartum. The proportion of women using contraceptives was highest (48.5%) in the first 3 months from delivery, little more than a quarter of women initiated contraceptive in 4–6 months, while about a quarter of them adopted it after 6 months. Female sterilization was the highest adopted contraceptive method in first trimester of postpartum period. While the use of foam or jelly was highest within 4–6 months from the time of delivery. Use of pill as a contraceptive pills and injection (37% & 36% respectively) was highest within 7–12 months after childbirth.

**Table 1:** Percentage of contraceptive use after having recent birth by postpartum Time Period, India, NFHS-2015-2016.

	Time Period			N
Modern Contraceptive Method	Within 3 months	4–6 months	7–12 months	
Pill	29.09	34.09	36.83	9,283
IUD	50.23	28.00	21.77	3,009
Injections	34.34	28.88	36.79	379
Diaphragm	64.06	0.00	35.94	3
Condom	37.64	37.27	25.09	13,274
Female sterilization	72.90	13.25	13.86	24,320
Male sterilization	49.37	31.03	19.6	165
Female condom	51.51	37.23	11.27	75
Foam or jelly	25.67	74.33	0.00	3
Other modern method	45.70	23.89	30.41	26
**Total**	**48.49**	**27.09**	**24.42**	**50,537 (37%)**

Table 2 shows the proportion of women adopting different contraceptive methods according to their pregnancy intentions within a year after recent birth. It was found that women who wanted the recent child majority adopted female sterilization (18%), while those stated no intention of more child, 20% adopted female sterilization as the sole contraceptive method, while 16% also relied on the modern spacing method of contraceptives. Among those who stated that they had an intent of child bearing, majority among them were found using male condom (14%), followed by female sterilization.

**Table 2:** Percentage of contraceptive use within one year after having recent birth by woman’s pregnancy intention, India, NFHS-2015-2016.

	Wanted Then	Wanted Later	Wanted no more
Not using	62.98	62.52	65.58
Pill	6.73	9.46	5.77
IUD	2.21	2.92	1.53
Injections	0.27	0.37	0.36
Diaphragm	0.00	0.02	0.00
Condom	9.72	13.18	6.96
Female sterilization	17.91	11.19	19.61
Male sterilization	0.12	0.29	0.09
Female condom	0.06	0.04	0.02
Foam or jelly	0.00	0.00	0.02
Other modern method	0.02	0.02	0.04

### Contraceptive initiation among currently married women with respect to socioeconomic variables and MCH care

Table 3 shows the percentage distribution of currently married women (aged 15–49) using contraceptive use according to socioeconomic and demographic variables and MCH indicators (after the birth of the last child). The gender composition of the living child/children significantly impacted contraceptive use. While 35% women who had single son used contraceptive within a year of recent birth, the uptake was relatively low among women with only child who was daughter (29%). The study hypothesized that using MCH indicators is pivotal in increasing contraceptive initiation. The percentage distribution of MCH indicators highlights the association. Among those women using contraceptive method within 12 months postpartum, 45% had 4+ ANC visits, 42% women received PNC within 41 days after the delivery, 44% women took IFA tablets for at least 100 days, 40% of women provided DPT-3 immunization to their child and 38% women received 2 tetanus injections.

**Table 3:** Percentage distribution of contraceptive use after having recent birth by MCH care and socio-demographic variables.

Maternal and Child care	Percentage
Number of Antenatal care (ANC) visits (At least 4)	44.62
Postnatal care (PNC) of mother (within 41 days after the delivery)	42.47
Number of Tetanus injections received during pregnancy (at least 2).	38.32
DPT-3 immunization received	39.69
Institutional delivery by trained professionals	38.84
100+ Iron Folic Tablets	44.33
**Place of Residence**	
Urban	42.99
Rural	34.19
**Region**	
North	45.5
Central	28.54
East	32.59
North East	35.52
West	41.83
South	44.44
**Religion**	
Hindu	36.74
Muslim	34.83
Others	45.80
**Caste**	
SC/ST	36.36
OBC	34.13
Others	42.25
**Economic status**	
Poorest	25.23
Poorer	34.85
Middle	39.50
Richer	42.00
Richest	46.14
**Age Group**	
<25	32.32
25–34	39.73
35–44	34.46
45+	18.63
**Education**	
No education	28.52
Primary	37.26
Secondary	40.51
Higher	41.97
**Parity of women**	
Less than 2 child	25.85
More than equal to 2 child	42.57
**Child Composition**	
Only Son	35.38
Only Daughter	29.34
Both	42.25
**Child Loss**	
No loss	37.84
1 loss	30.68
2 loss	25.49
**Mass media Exposure**	
No	36.84
Yes	38.46
**Wanted Last child**	
Wanted	37.02
Wanted later	37.48
Never wanted	34.42

Note- for chi-square analysis all the variables were found to be significant, expect for child’s wanted status, but was included in the multivariate model, because of relative importance from literatures.

### Multivariable complementary log-log regression analysis

The utilization of MCH care varies according to the background characteristics of women. So, we have presented the correlation coefficient of MCH index by different socio-demographic variables for better understanding as MCH index is directly associated with family planning use. We found all the variables were significant (S3 Table).

The likelihood of adopting modern contraceptive varies with the gender composition of children. The chances of contraceptive adoption were higher when women had a male child compared to those who had only daughters (AOR = 1.153,95% CI– 1.222, 1.184). Women who suffered more than two child loss are less likely to adopt modern contraceptive than those who never suffered a child loss (AOR = 0.890, 95% CI- 0.807–0.980). The loss of child could be a result of failure to access or adopt the contraceptive in timely manner and therefore the child loss could prompt them to learn and adopt the methods actively.

MCH index was found to have a significant association with early initiation of contraceptive methods. Women who utilized MCH services were 1.08 times more likely to adopt contraception after delivery (Table 4). A plausible explanation for this association could be that women using health services are more likely to come in contact with the health workers and, in turn, more likely to gain knowledge on different family planning methods which encourage and equip them to adopt various contraceptive methods.

**Table 4:** Discrete time complementary log -log model results on modern contraceptive adoption by selected covariates, NFHS, 2015–2016.

Covariates	AOR (95% CI)
**Place of Residence**	
Urban	1.00
Rural	0.964*(0.926 1.004)
**Region**	
North	1.00
Central	0.860***(0.829 0.892)
East	0.920***(0.881 0.961)
North East	0.770***(0.734 0.808)
West	1.115***(1.057 1.176)
South	2.525***(2.380 2.680)
**Religion**	
Hindu	1.00
Muslim	0.927***(0.883 0.973)
Others	0.961 (0.902 1.024)
**Caste**	
SC/ST	1.00
OBC	0.953**(0.918 0.99)
Others	1.015 (0.973 1.059)
**Economic status**	
Poorest	1.00
Poorer	1.137***(1.092 1.184)
Middle	1.198***(1.144 1.255)
Richer	1.240***(1.175 1.310)
Richest	1.285***(1.201 1.375)
**Age Group**	
<25	1.00
25–34	0.963**(0.928 0.999)
35–44	0.908***(0.848 0.972)
45+	0.715***(0.615 0.832)
**Education**	
No education	1.00
Primary	1.083***(1.031 1.137)
Secondary	1.082***(1.035 1.133)
Higher	1.026 (0.958 1.099)
**Parity of women**	
Less than 2 child	1.00
More than equal to 2 child	1.876***(1.793 1.962)
**Child Composition**	
Only Daughter	1.00
Only Son	1.153***(1.122, 1.184)
Both	1.149***(1.11 8 1.181)
**Child Loss**	
No loss	1.00
1 loss	1.027 (0.982 1.073)
2 loss	0.890**(0.807 0.98)
**Mass media Exposure**	
No	1.00
Yes	0.930 (0.844 1.024)
**MCH Index**	1.079***(1.06 1.098)

## Discussion

India was the first country to launch its own family planning programme and population policy in 1952, the primary concern of the plan was to achieve population stabilization by lowering fertilization rates [32]. In 1971, the focus shifted to population control, and mass sterilization took place in the country [33]. Since then, there has been no significant improvement in the unmet need for family planning [28, 34]. Many studies have been conducted on trends, patterns, awareness differentials of contraceptive use in India [32, 35], and some studies have tried to explore the determinants of utilization and non-utilization of contraceptives. However, this study found that MCH services were significantly associated with early initiation of contraceptive among women within 12 months of the childbirth.

Out of a sample population of 1,36,962 women, only 37% of women initiated modern contraceptive methods within 12 months of childbirth. After controlling for several socioeconomic and demographic factors in our model, we identified several determinants for the adoption of contraceptives. The region, place of residence (urban or rural) of women, religion, caste, wealth index of the households where women live, women’s age, desire for one more child emerged as the most significant explanatory factors for early initiation of contraception. Early adoption of contraceptives was higher in South India while least in Central India. These findings corroborate with findings of a study conducted in Andhra Pradesh, a South Indian state, which tops among all the states in adoption of modern contraceptive [36]. The urban respondents adopt modern contraceptives earlier than their rural counterparts, which is similar to the findings from another study [9]. The view on birth control varies among religions. A lag in the early adoption of contraceptives was found among non-Hindu women. Among the major religions, Islam and Christianity have prenatal opinions, which could be a potential reason for the lagging in early adoption of contraception among non-Hindu women [37]. The odds of early contraceptive adoption were higher in women at 25–34 years of age than older women as sexual activity, and coital frequency are higher among young couples to achieve the desired number of children [31]. Women’s education was not significantly associated with the early adoption of contraceptives, contrary to what was observed in other studies [9]. Similar to the findings from other studies, women belonging to poor households have a lower chance of adopting the contraceptive method than those from wealthy families [38]. The loss of one or more children was associated with lower likelihood of adopting contraceptive similar to findings from studies conducted in Bangladesh and Iran [39]. There was a significant association between the sex composition of living children and reproductive factors such as parity progression and contraceptive use [9, 40–43]. Those who never wanted the last-born child had higher odds of early adoption of the contraceptive, similar to the findings from other studies [9]. Exposure to both print and electronic media increases the odds of early initiation of contraception. Our results corroborate with the findings of the studies from Pakistan, Bangladesh, and Indonesia [44].

The most important finding of this study is that women with higher uptake of MCH services have a higher likelihood of early contraceptive adoption. Several studies which have explored the relationship between various MCH services and contraceptive initiation have found a positive association [24]. A recent study based on population surveys in Kenya and Zambia concluded that the intensity of ANC and PNC services are positively associated with modern contraceptive initiation [5, 45]. The findings from an international study that constructed an index comprising of factors on ANC, PNC, delivery care, and child vaccination suggested that MCH services could serve as a ’gateway’ for promoting family planning in Guatemala, Morocco, and Indonesia [46]. The plausible explanations for this association could be that the women who use MCH services are more likely to be exposed to family planning services. Also, a woman may develop trust over time with the healthcare system overall, encouraging her to access several services from the system. A woman’s early contact with the health care system reduces the psychosocial, cognitive, and indirect financial barrier (in the form of opportunity cost and time). This makes subsequent contact with the system for family planning services easier.

Though the study provided insights on the relationship of MCH care and subsequent contraceptive use among currently married women in 12 months of postpartum in India, there are few limitations. First of all, the analysis is based on observational data. Second, our study doesn’t adjust for hierarchical structure and assumes that the population is homogenous. Whereas a study adjusting for individual and community level factors, clustering (frailty) found dose response type of relationship between maternal health care services and the time of use of contraceptive after childbirth [47]. Also, in this study, we have only considered women who could avail MCH services. However, several women couldn’t avail the MCH services due to unavailability of services about which the NFHS doesn’t provide information. The decision on using maternal and modern family planning methods can be influenced by several factors such as insufficient contraceptive method mixes, and poor integration of family planning service with other health services, which are not controlled for in this study.

## Conclusion

The proportion of women receiving four or more ANC visits has increased from 37% (NHFS-3) to 51% (NFHS-4). Similarly, 65% of women received PNC in the first two days after birth from 37% in 2005–06. As the postpartum contraceptive initiation is associated with ANC, PNC factors that are delivered through the healthcare system, providing family planning services alongside MCH services will prove to be cost-effective and efficient in low resource settings. Our study findings support and add to the existing literature on the association of MCH indicators and postpartum contraceptive adoption that despite the considerable improvement in all other MCH indicators; the early initiation of contraceptive during the postpartum period remains poor. And this could be of vital importance for family planning programmes targeted at improving contraceptive adoptions. As per the NHFS-4 report, the proportion of women completing at least one or more ANC has increased considerably over the last decade. It is an invaluable opportunity to educate and encourage women to adopt contraceptive early. In a pilot study conducted in Zambia, focused MCH packages improve the experiences of clients and health providers and significantly improve the overall quality of care [48]. This makes women feel more comfortable, and they are more likely to approach the MCH health system when in need. As highlighted in the limitations of the study, it is possible that community-level norms or access to family planning practices could influence an individual’s behaviour of contraceptive uptake. But, some of these data were not readily available with the DHS survey and hence couldn’t be included. Hence, this elicits the need for future research and further studies to understand the reasons behind poor postpartum contraceptive adoption among women in their reproductive ages.

## Supporting information

S1 Table Description of explanatory factors. (DOCX)

S2 Table Descriptive statistics of variables. (DOCX)

S3 Table Correlation coefficient of MCH index by socio-demographic variables. (DOCX)

## Abbreviations

MCH: Maternal and Child Health Care
MMR: Maternal Mortality rate
IMR: Infant Mortality rate
SC/ST: Scheduled Caste and Tribe
DPT: Diphtheria-pertussis-tetanus
MoHFW: Ministry of Health and Family Welfare
