# Supplementary material for: Role of maternal and child health services on the uptake of contraceptive use in India: A reproductive calendar approach
Source: PLoS One. 2022 Jun 15;17(6):e0269170. doi: 10.1371/journal.pone.0269170 (PMC9200305; doi:10.1371/journal.pone.0269170)
Supplement: S1 Table — (DOCX) [file pone.0269170.s001.docx]

**Table S1: Description of Explanatory factors**

| **Variables** | **Categories** |
| --- | --- |
| **Residence** | Urban, Rural |
| **Regions of India** | North, Central, East, North-East, West, South |
| **Religion** | Hindu, Muslim, Others |
| **Caste** | SC/ST, OBC, Others |
| **Economic Status** | Richest, Rich, Middle, Poorer, Poor |
| **Age group (women)** | >25, 25-34,35-44.45+ |
| **Education** | No Education, Primary, Secondary, higher |
| **Characteristics of the Child** |  |
| **Child dead** | No child,1 child,2+male |
| **Parity of women** | Less than 2 child, more than equal to 2 child |
| **Child composition** | Only son, only daughter, both |
| **Wanted child** | Wanted, Wanted later, not Wanted |
| **Mass media Knowledge** | Yes, No |
