# Supplementary material for: Role of maternal and child health services on the uptake of contraceptive use in India: A reproductive calendar approach
Source: PLoS One. 2022 Jun 15;17(6):e0269170. doi: 10.1371/journal.pone.0269170 (PMC9200305; doi:10.1371/journal.pone.0269170)
Supplement: S2 Table — (DOCX) [file pone.0269170.s002.docx]

**Table S2: Descriptive Statistics of variables**

|  | **N** | **Percentage** |
| --- | --- | --- |
| **Maternal and Child care (Mean and S.E)** | 0.09 (0.005) | |
| Number of Antenatal care (ANC) visits (At least 4 visits). | 70,534 | 51.50 |
| Postnatal care (PNC) of mother (within 2 weeks after the delivery). | 49,084 | 35.84 |
| Number of Tetanus injections received during pregnancy (at least 2). | 1,13,987 | 83.23 |
| DPT-3 immunization received. | 1,04,804 | 76.52 |
| Institutional delivery by trained professionals. | 1,10,482 | 80.67 |
| 100+ Iron Folic Tablets | 41,642 | 30.40 |
| **Place of Residence** |  |  |
| Urban | 42,156 | 30.78 |
| Rural | 94,806 | 69.22 |
| **Region** |  |  |
| North | 18,028 | 13.16 |
| Central | 34,019 | 24.84 |
| East | 34,940 | 25.51 |
| North-East | 5,513 | 4.03 |
| West | 18,310 | 13.37 |
| South | 26,152 | 19.09 |
| **Religion** |  |  |
| Hindu | 1,07,901 | 78.78 |
| Muslim | 22,065 | 16.11 |
| Others | 6,996 | 5.11 |
| **Caste** |  |  |
| SC/ST | 42,229 | 30.83 |
| OBC | 59,598 | 43.51 |
| Others | 35,135 | 25.65 |
| **Economic status** |  |  |
| Poorest | 31,384 | 22.91 |
| Poorer | 28,328 | 20.68 |
| Middle | 27,029 | 19.73 |
| Richer | 26,592 | 19.42 |
| Richest | 23,628 | 17.25 |
| **Age Group** |  |  |
| <25 | 39,691 | 28.98 |
| 25-34 | 82,242 | 60.05 |
| 35-44 | 14,096 | 10.29 |
| 45+ | 933 | 0.68 |
| **Education** |  |  |
| No education | 38,199 | 27.89 |
| Primary | 18,597 | 13.58 |
| Secondary | 63,818 | 46.60 |
| Higher | 16,348 | 11.94 |
| **Parity of women** |  |  |
| Less than 2 child | 46,475 | 33.93 |
| More than equal to 2 child | 90,486 | 66.07 |
| **Child Composition** |  |  |
| Only Son | 40,547 | 29.60 |
| Only daughter | 35,219 | 25.71 |
| Both | 61,195 | 44.68 |
| **Child Loss** |  |  |
| No loss | 1,21,305 | 88.57 |
| 1 loss | 12,537 | 9.15 |
| more than 2 loss | 3,119 | 2.28 |
| **Mass media Knowledge** |  |  |
| Yes | 1,31,627 | 96.11 |
| No | 5,334 | 3.89 |
| **Wanted Last child** |  |  |
| Wanted | 1,24,570 | 90.95 |
| Wanted later | 4,949 | 3.61 |
| Never wanted | 7,442 | 5.43 |
