# Supplementary material for: Role of maternal and child health services on the uptake of contraceptive use in India: A reproductive calendar approach
Source: PLoS One. 2022 Jun 15;17(6):e0269170. doi: 10.1371/journal.pone.0269170 (PMC9200305; doi:10.1371/journal.pone.0269170)
Supplement: S3 Table — (DOCX) [file pone.0269170.s003.docx]

**Table S3: Correlation coefficient of MCH index by socio-demographic variables**

|  | **Correlation Coefficient** |
| --- | --- |
| **Place of Residence** |  |
| Urban | 0.18 |
| Rural | -0.18 |
| **Region** |  |
| North | 0.05 |
| Central | -0.17 |
| East | -0.10 |
| North-East | -0.01 |
| West | 0.13 |
| South | 0.22 |
| **Religion** |  |
| Hindu | -0.01 |
| Muslim | 0.01 |
| Others | 0.01 |
| **Caste** |  |
| SC/ST | -0.07 |
| OBC | -0.03 |
| Others | 0.11 |
| **Economic status** |  |
| Poorest | -0.26 |
| Poorer | -0.08 |
| Middle | 0.06 |
| Richer | 0.13 |
| Richest | 0.20 |
| **Age Group** |  |
| <25 | 0.00 |
| 25-34 | 0.04 |
| 35-44 | -0.05 |
| 45+ | -0.04 |
| **Education** |  |
| No education | -0.26 |
| Primary | -0.05 |
| Secondary | 0.17 |
| Higher | 0.16 |
| **Parity of women** |  |
| Less than 2 child | 0.14 |
| More than equal to 2 child | -0.14 |
| **Child Composition** |  |
| Only Son | 0.08 |
| Only daughter | 0.08 |
| Both | -0.14 |
| **Child Loss** |  |
| No loss | 0.10 |
| 1 loss | -0.08 |
| 2 loss | -0.07 |
| **Mass media Exposure** |  |
| No | -0.08 |
| Yes | 0.08 |
| **Wanted Last child** |  |
| Wanted | 0.08 |
| Wanted later | -0.01 |
| Never wanted | -0.09 |

Note-all the variables were found significant.
